# Longitudinal assessment of the exposure to *Ascaris lumbricoides* through copromicroscopy and serology in school children from Jimma Town, Ethiopia

**DOI:** 10.1371/journal.pntd.0010131

**Published:** 2022-01-18

**Authors:** Daniel Dana, Sara Roose, Johnny Vlaminck, Mio Ayana, Zeleke Mekonnen, Peter Geldhof, Bruno Levecke

**Affiliations:** 1 School of Laboratory Science, Faculty of Health Science, Institute of Health Jimma University, Jimma, Ethiopia; 2 Department of Translational Physiology, Infectiology and Public Health, Ghent University, Merelbeke, Belgium; Seoul National University College of Medicine, REPUBLIC OF KOREA

## Abstract

**Background:**

We previously demonstrated that serology holds promise as an alternative diagnostic tool to copromicroscopy to monitor and evaluate deworming programs targeting soil-transmitted helminths (STHs). Here we explored the dynamics of anti-*Ascaris* antibodies (Ab) and evaluated the Ab-isotype of choice to assess the longitudinal exposure to *Ascaris* in Ethiopian school children.

**Methodology:**

Between October 2018 and February 2020, stool and blood samples were collected every four months from school children (4 to 6 years of age). Stool samples were analyzed by duplicate Kato-Katz to assess the presence and intensity of any STH infection. Plasma Ab-responses against the total extract of *Ascaris suum* lung third stage larvae were measured through in-house Ab-ELISA’s for seven different Ab-isotypes.

**Principal findings:**

At baseline, 42.4% of the 66 children were excreting eggs of any STH, *Trichuris* (37.9%) being the most prevalent. The cumulative prevalence (proportion of children tested that positive at least once over the entire study period) was 56.1% for *Trichuris* and 31.8% for *Ascaris*. For *Ascaris*, re-infections were frequently observed, whereas for *Trichuris*, children often remained excreting eggs following drug administration. When measuring anti-*Ascaris* Ab-levels, the cumulative seroprevalence was generally higher (IgG4: 60.6%; IgG1: 50.0%; IgE: 36.4%). The individual anti-*Ascaris* IgG4 levels at baseline were positively associated with the fecal egg counts averaged over the study period, the rate of egg-appearance and the number of positive test results. There was no apparent cross-reactivity between the anti-*Ascaris* IgG4 Ab-ELISA and *Trichuris*.

**Conclusions/Significance:**

We demonstrate that the children are exposed to STH before the age of four and that the exposure to *Ascaris* is underestimated when measured with copromicroscopy. Compared to other Ab-isotypes, IgG4 is the Ab-isotype of choice to measure *Ascaris* exposure in STH endemic settings. Finally, the results also highlight that measuring anti-*Ascaris* IgG4 levels holds promise as a tool to identify individuals at higher risk for continued exposure to this STH.

## Author summary

World-wide large-scale deworming programs are being implemented to control the morbidity attributable to intestinal worms. Our group has recently shown that diagnostic tools that detect anti-parasite antibodies hold promise as an alternative to the current diagnostic standard (detecting worm eggs in stool using microscopy) to monitor and evaluate the progress of these programs towards the set targets. In the present study, we further explored the potential of antibody-based assays by monitoring the exposure of Ethiopian school children during a 17-month period to worm infections. Our results indicated that children in this area are exposed to intestinal worms from an early age onwards and that, based on serological results, more children are exposed to worm infections than measured by the current diagnostic standard. In addition, we demonstrated that test results of the antibody assay at the start of the study were positively associated with mean egg counts averaged over the study period, the rate of egg-appearance and the number of positive stool tests, highlighting that such assay holds promise as a tool to identify individuals at higher high risk for continued exposure to worms, and ultimately improve control programs.

## Introduction

The soil-transmitted helminths (STHs) are a group of intestinal worms including *Ascaris lumbricoides*, *Trichuris trichiura* and the hookworm species *Necator americanus Ancylostoma duodenale* and *Ancylostoma ceylanicum* [1]. In 2019, it was estimated that globally 909 million people were infected with one of these four STHs and that they accounted for a loss of 1.97 million disability adjusted life years (DALYs) [2,3]. To fight the STH-attributable morbidity, the World Health Organization (WHO) recommends large scale-deworming programs in which anthelmintic drugs are periodically administered to at-risk populations living in endemic areas (e.g. children and women of child bearing age). Recently, WHO published six ambitious targets for soil-transmitted helminthiasis by 2030, of which monitoring of the first two will be based on large-scale epidemiological surveys to reduce the prevalence of moderate-to-heavy intensity of any STH infections in children to less than 2% in all endemic countries (**Target #1**) and to reduce the global need for anthelmintic tablets in large scale deworming programs by 50% (**Target #2**) [4].

Although Kato-Katz thick smear remains the diagnostic standard in the WHO 2030 roadmap, the debate is still ongoing whether this stool-based test is the most appropriate diagnostic tool to monitor progress towards the two aforementioned program targets [4]. Indeed, to fill this gap of improved diagnostic tests, the STH community has been engaged in describing the minimal and ideal characteristics (e.g. sample type, clinical sensitivity and specificity, cost per sample and number of samples analysed per hour per person)—the so-called target product profiles (TPPs) [5–8]. These TPPs highlight an apparent shift in interest towards non-stool based tests (e.g. serum/plasma and urine samples), as they would simplify the sample collection process, minimize the operator variability and increase the sample throughput.

Faced with similar challenges in veterinary medicine, our group has developed two antibody-ELISAs (Ab-ELISA) to measure exposure to *Ascaris suum* infections in pigs [9–11]. These tests are based on the *Ascaris suum* haemoglobin (AsHb) from adult worms and the total extract of *Ascaris suum* lung third stage larvae (AsLungL3) and have proven to be more sensitive than the current stool-based diagnostic standard in veterinary medicine (McMaster egg counting method [12]). Both Ab-ELISAs have also been field tested in human populations in Indonesia (AsHb) [13] and Ethiopia (AsHb and AsLungL3) [14], highlighting that the AsLungL3 extract contains promising antigens for further diagnostic tool development. For the AsLungL3 Ab-ELISA, we demonstrated a reduction in exposure (measured by seroprevalence and/or mean optical density (OD)) in Ethiopian school children following multiple rounds of a large-scale school-based deworming program [14]. In addition, we demonstrated in the same endemic area that adults are continuously exposed to the infectious stages of *Ascaris*, a finding that was so far not observed for stool examination [14].

However, currently we have few insights into the longitudinal *Ascaris* exposure dynamics in children when measured by both copromicroscopy and Ab-ELISA, with ‘exposure’ being defined as exposed to parasite eggs, larvae and/or adults. Therefore, we followed a cohort of school children in an STH endemic setting in Ethiopia over a period of 17 months with the aim to assess and compare the longitudinal *Ascaris* exposure dynamics when measured by Kato-Katz thick smear and the AsLungL3 Ab-ELISAs for different Ab-isotypes.

## Methods

### Ethics statement

The study protocol was approved by the institutional review board of the Institute of Health, Jimma University (Ref N°: IHRPGD/681). The objectives and procedures of the study were disclosed to children and both their teachers and parents/guardians. A written informed consent was prepared in the two commonly used languages in the Jimma Town (Afaan Oromo and Amharic). Only those children who had a signed written informed consent from their parents/ guardians and orally assented to participate in the study were included. One copy of the signed consent was handed over to the children’s parents/guardians. A schedule for the different follow-up visits was developed and provided to both parents and the children. The written informed consent and assent was repeated at follow-up visits. Based on the test results of the stool examination, all children with any STH infection (presence of any STH egg in stool) were treated with a single oral dose of albendazole (400 mg) after each positive test result. On top of this, the children were also treated bi-annually according to the national deworming program in the area. Children infected with other intestinal helminths (e.g. *Taenia* spp, *Hymenolepis nana*, *Enterobius vermicularis* and *Schistosoma mansoni)* were referred to the local health centres for further treatment. Blood samples from the non-endemic adult population in Belgium were acquired through the Red Cross Flanders (order N°: CG2016 0404A, CM2016 0627B and CG2016 1219F).

### Study design

**Fig 1:**
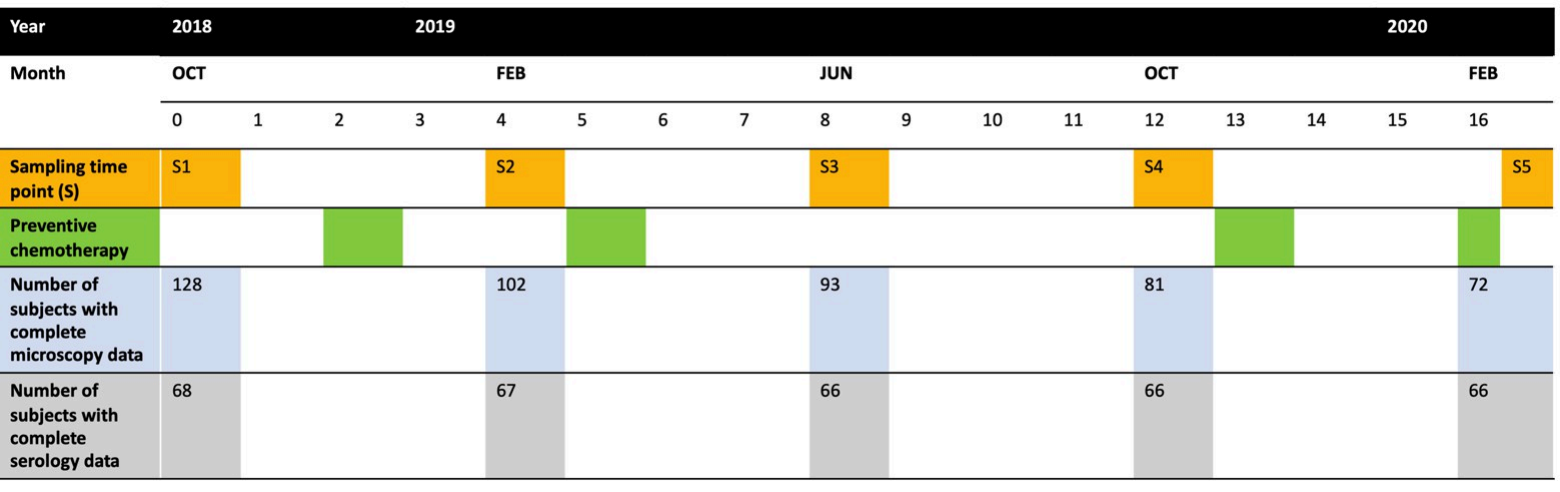
Time of sampling, preventive chemotherapy and number of participating subjects. The timeline provides an overview of the different 4-monthly sampling points (S1–5) between October 2018 and October 2020, the administration of a single oral dose of albendazole as part of national preventive chemotherapy program and the number of subjects for whom microscopic data was available. We prematurely stopped the study due to the COVID-19 pandemic. As a consequence of this, no subjects were screened during the last two sampling points (June and October 2020). Test results for both microscopic examination and Ab-ELISA (for all Ab-isotypes) throughout the study was available for 66 subjects.

Between October 2018 and February 2020, we followed a cohort of school children of one school in Jimma Town (Ethiopia). The selection of the school was based on its previous involvement in epidemiological [15] and drug efficacy studies [16,17], and the apparent high prevalence of STH infections [14,18,19]. At baseline, a total of 128 school children aged 4 to 6 years were included. Every four months, both a stool and a blood sample were collected from each selected child at the school. To this end, we provided the parents/guardians of the children a clean and capped stool container with a unique identification. The parents/ guardians were instructed to collect about 5 g stool of their child. From those children who provided sufficient stool, we collected 100 to 200 μL capillary blood using a sterile lancet and capillary blood collection tube. All samples were placed in cool box for transport. Upon arrival at the Neglected Tropical Disease Laboratory of Jimma University, all samples were immediately processed. We applied a duplicate Kato-Katz thick smear on each stool sample and recorded the fecal egg counts (FECs; expressed as eggs per gram of stool (EPG)) as described in our previous work [14]. The intensity of infection was determined based on the FEC thresholds recommended by WHO [20]. We separated the plasma from the heparinised capillary blood and stored it at -20°C until the samples were shipped to the Laboratory of Parasitology, Ghent University. At Ghent University, all plasma samples were subjected to the AsLungL3 Ab-ELISAs to detect anti-*Ascaris* IgA, IgE, IgM and IgG sub-classes (IgG1, IgG2, IgG3 and IgG4). The cut-offs that define a positive test result were determined by re-examination of a subset of blood samples from 91 healthy Belgian blood donors. The samples collected from the school children were considered positive when the OD was at least equal to the mean OD observed in the samples from the healthy Belgian blood donors + 3 times the corresponding standard deviation. **Fig 1** provides an overview of the different sampling periods, the rounds of national deworming and the participation rate over time, and the number of children incorporated in the final data analysis.

### Ab-ELISA

Plasma samples were analysed on an Ab-ELISA test based on a water-soluble complete homogenate from L3 larvae migrating through the lungs. The homogenate was produced as described by Vlaminck et al., 2016 [21]. In brief, piglets were infected with approximately 200,000 infective *A*. *suum* eggs and sacrificed 7 days post-infection. The lung L3 were collected from minced lung tissue that was placed on a modified Baermann device. Larvae were washed excessively using PBS at 4°C and stored at -80°C. L3 protein extracts were prepared by grinding the collected larvae with a mortar and pestle that was placed in a bath of liquid nitrogen. The larval homogenate was mixed with PBS and proteinase inhibitor cocktail (1:100) (Sigma). The homogenate was then inverted at 4°C for 2 hours followed by centrifugation for 10 min at 31,500 *g* at 4°C. The supernatant (AsL3Lung extract) was removed and stored at -80°C.

Serological analysis of the plasma samples was performed based on the protocol described by Dana et al., 2020 [14]. In short, Maxisorp 96-well ELISA plates were coated overnight at 4°C with 5 μg/mL of AsL3Lung extract in carbonate buffer (pH = 9.6). After three wash steps with wash buffer (PBST: phosphate buffered saline containing 0.05% Tween20), non-specific binding sites were blocked for 2 hours at 4°C using blocking buffer (PBS + 5% heat treated fetal calf serum). The coated antigens were detected by adding diluted sera at a 1/100 to 1/400 dilution (optimal dilution was defined for each antibody isotype separately) in PBST for 2 hours at room temperature. Following three subsequent wash steps, horseradish peroxidase conjugated anti-human IgG1, IgG2, IgG3, IgG4, IgM, IgA or IgE in blocking buffer (optimal dilution was again defined for each conjugate separately) was added to the plate for 1 hour at room temperature. The details on these secondary antibodies are summarized in **S1 Info**. After a final three washing steps with PBST, 100 μL of substrate was added (O-phenylenediamine 0.1% in citrate buffer (pH = 5.0)). The development reaction was stopped after 10 min with 2.5M HCl and the OD was measured at 492 nm.

### Statistical data analysis

We reported the cumulative prevalence over the different sample points (proportion of children that tested positive at least once in a given time interval) and the incidence (the proportion of the subjects that tested negative at baseline but that tested positive at least once in a given time interval) for both Ab-ELISAs and copromicroscopy. In addition, individual FECs (in EPG) and Ab-responses (in OD) were plotted to gain insights into the infection and Ab-response dynamics, respectively. Given the high number of IgG4 seropositive children at baseline, we also investigated whether this parameter could be used as a predictor for *Ascaris* egg excretion later on. For this, children were categorized into three categories based on their anti-*Ascaris* IgG4 levels at baseline, i.e. no Ab-response: OD < diagnostic cut-off; low Ab-response: diagnostic cut-off ≤ OD < median OD across all children tested positive for this Ab-isotype; high Ab-response: OD ≥ median OD across all children tested positive for this Ab-isotype. We verified whether the individual anti-*Ascaris* IgG4 levels at baseline were positively associated with the probability of both (i) excreting eggs at baseline and (ii) testing positive at least two times during the study period (odds ratio), and (iii) the FEC averaged over the study period (Spearman correlation coefficient (Rs)). In addition, the rate of egg-appearance (change of cumulative prevalence over time) was assessed. Finally, these associations between anti-*Ascaris* IgG4 levels at baseline and copromicroscopy, were also assessed for *Trichuris*, to assess any cross-reactivity between the anti-*Ascaris* IgG4 Ab-ELISA and *Trichuris*. All the data is made available in **S1 Dataset**. All statistical analyses were performed in R version 3.6.0 [22].

## Results

### Demographics of study population

A total of 128 school children were enrolled in this study, including 59 girls and 69 boys (sex ratio of girls to boys = 1:1.2). At the baseline, the age of the children ranged from 4 (26.6%) to 6 years (34.4%). The study was prematurely stopped due to the COVID-19 pandemic, resulting in five instead of the seven anticipated sample collection time points. The results for both stool and plasma examination were available for a total of 66 (51.6%) out of the 128 children that were initially enrolled. Overall, the boy to girl sex ratio was 1:0.8 with an age distribution of 19.7% of 4-years-olds, 43.9% of 5-years-olds and 36.4% of 6-years-olds.

### STH infection dynamics based on copromicroscopy

**Fig 2:**
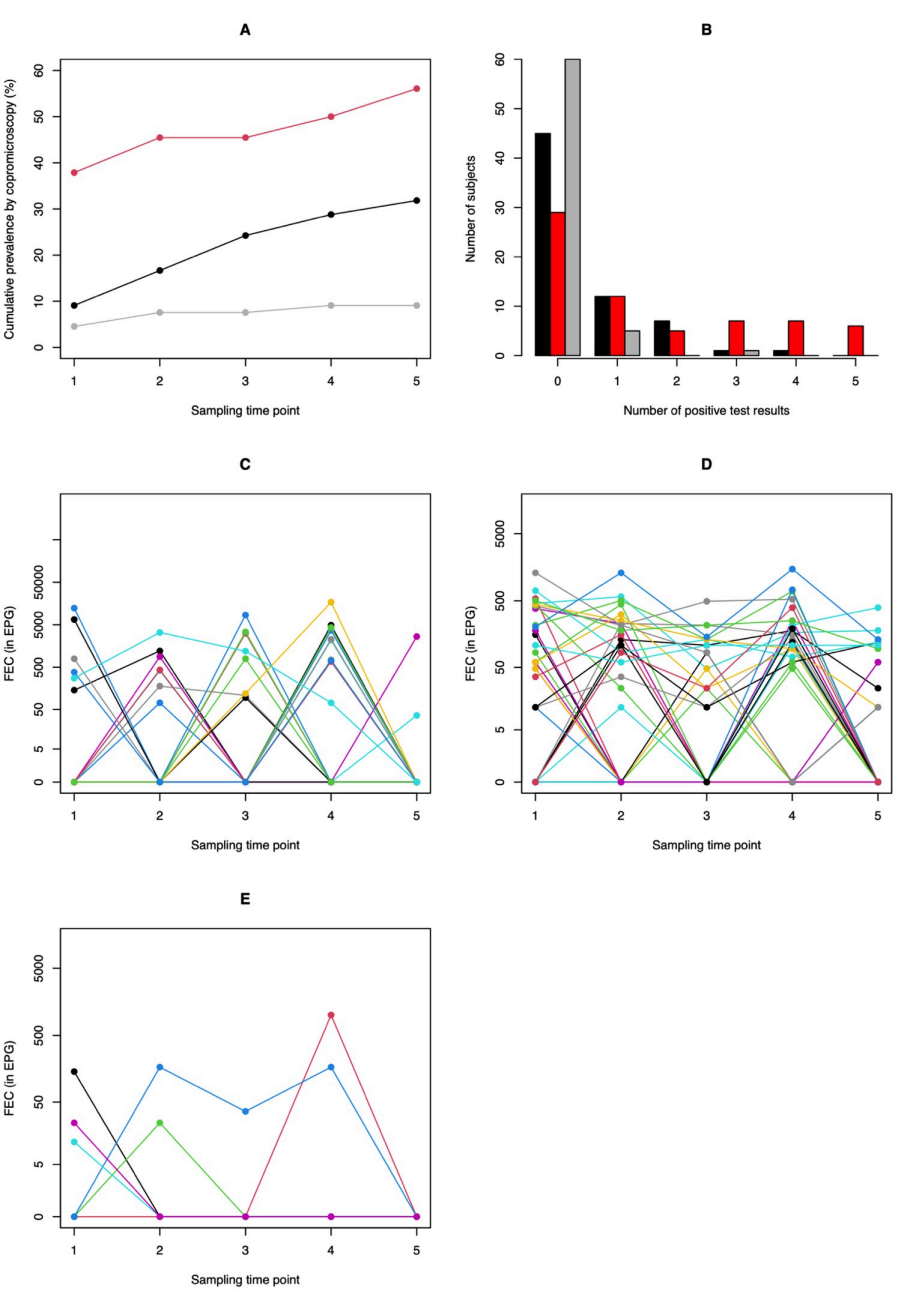
Soil-transmitted helminth infection dynamics based on duplicate Kato-Katz thick smear. **Panel A** illustrates the cumulative prevalence and **Panel B** the number of positive test results for each of the three STH species (*Ascaris*: black, *Trichuris*: red and hookworms: grey). The individual fecal egg counts (FECs; expressed as eggs per gram of stool) for *Ascaris*, *Trichuris* and hookworms over the five sample time points are shown in **Panels C**, **D** and **E**, respectively. For ethical reasons, children were treated when they were positive on copromicroscopy. Large scale deworming was implemented between each sampling time points, except between sampling time point 3 and 4 (**see Fig 1**).

The STH infection dynamics based on copromicroscopy across the 66 children are summarized in **Fig 2**. At baseline, at least one STH infection was observed in 42.4% of the children, with *T*. *trichiura* (37.9%) being the most prevalent STH species. Infections with *Ascaris* and hookworm were observed in 9.1% and 4.5% of the children, respectively. In the majority of the cases, the infection was low in intensity (*Ascaris*: 66.7%; *Trichuris*: 96.0% and hookworms: 100%). Over the entire study period, the number of *Ascaris*-infected individuals increased by a factor 3 (from 9.1% to 31.8%), while the cumulative prevalence of hookworms doubled (from 4.5% to 9.1%). The cumulative *Trichuris* prevalence increased from 37.9% to 56.1% (**Fig 2A**). The incidence based on copromicroscopy for each of the different STH species over the study period was 25.0% (15 out of 60) for *Ascaris*, 41.4% (12 out of 29) for *Trichuris*, and 4.8% (3 out of 63) for hookworms.

As shown in **Fig 2B**, most children were not continuously excreting eggs of STHs. Out of the 37 children excreting *Trichuris* eggs on at least one sample time point, 25 tested positive (67.6%) on at least two sampling time points. This was the case for 9 out of 21 subjects (42.9%) for *Ascaris* and 1 out of 6 subjects (16.7%) for hookworms. The differences between the STH species are also visible in the individual test results, as shown in **Fig 2C** (*Ascaris*)**, 2D** (*Trichuris*) and **2E** (Hookworm). While several children excreted *Trichuris* eggs at consecutive sampling time points (**Fig 2D**), the individual test results for *Ascaris* mostly shifted from a positive egg count to a zero-egg count (and vice versa) between two consecutive sample time points (**Fig 2C**). The individual test results for hookworms (**Fig 2E**) appeared more similar to *Ascaris* than *Trichuris*. Indeed, most children excreting eggs tested negative following a single dose of albendazole, only one child continued excreting eggs over three sampling time points.

### Dynamics of the anti-*Ascaris* antibody responses

**Fig 3:**
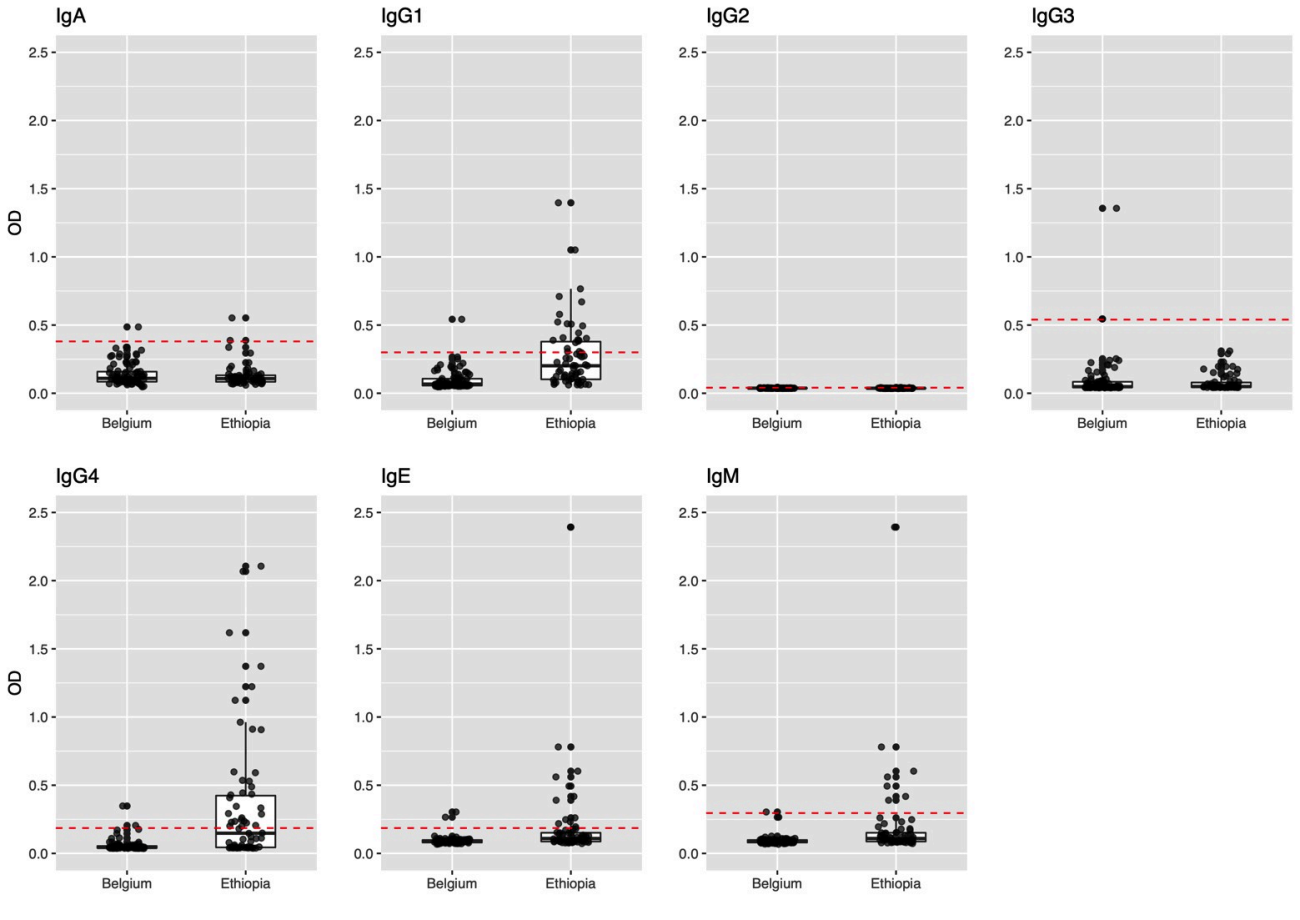
Anti-AsLungL3 antibody levels in a non-endemic and endemic area. Serum samples from 91 healthy adult Belgian blood donors and plasma samples of the 66 Ethiopian school children at the start of the study were analysed for anti-*Ascaris* IgA, IgG1, IgG2, IgG3, IgG4, IgE and IgM levels. The red dashed horizontal line indicates the diagnostic cut-off, which was defined as the mean optical density (OD) observed in the samples from the healthy Belgian blood donors + 3 times the corresponding standard deviation.

The results of the AsLungL3 Ab-ELISA’s on 91 healthy adult Belgian blood donors and the 66 Ethiopian school children at baseline are shown in **Fig 3**. Based on this threshold, the analysis indicated that the OD-profiles of the Belgian samples and the Ethiopian samples at baseline were almost similar for IgA, IgG2 and IgG3, whereas for the remaining Ab-isotypes (IgG1, IgG4, IgE and IgM), higher antibody levels were apparent in the baseline samples from Ethiopia, with 47.0% of the children testing positive for IgG4, 31.8% for IgG1 and 18.2% for IgE.

**Fig 4:**
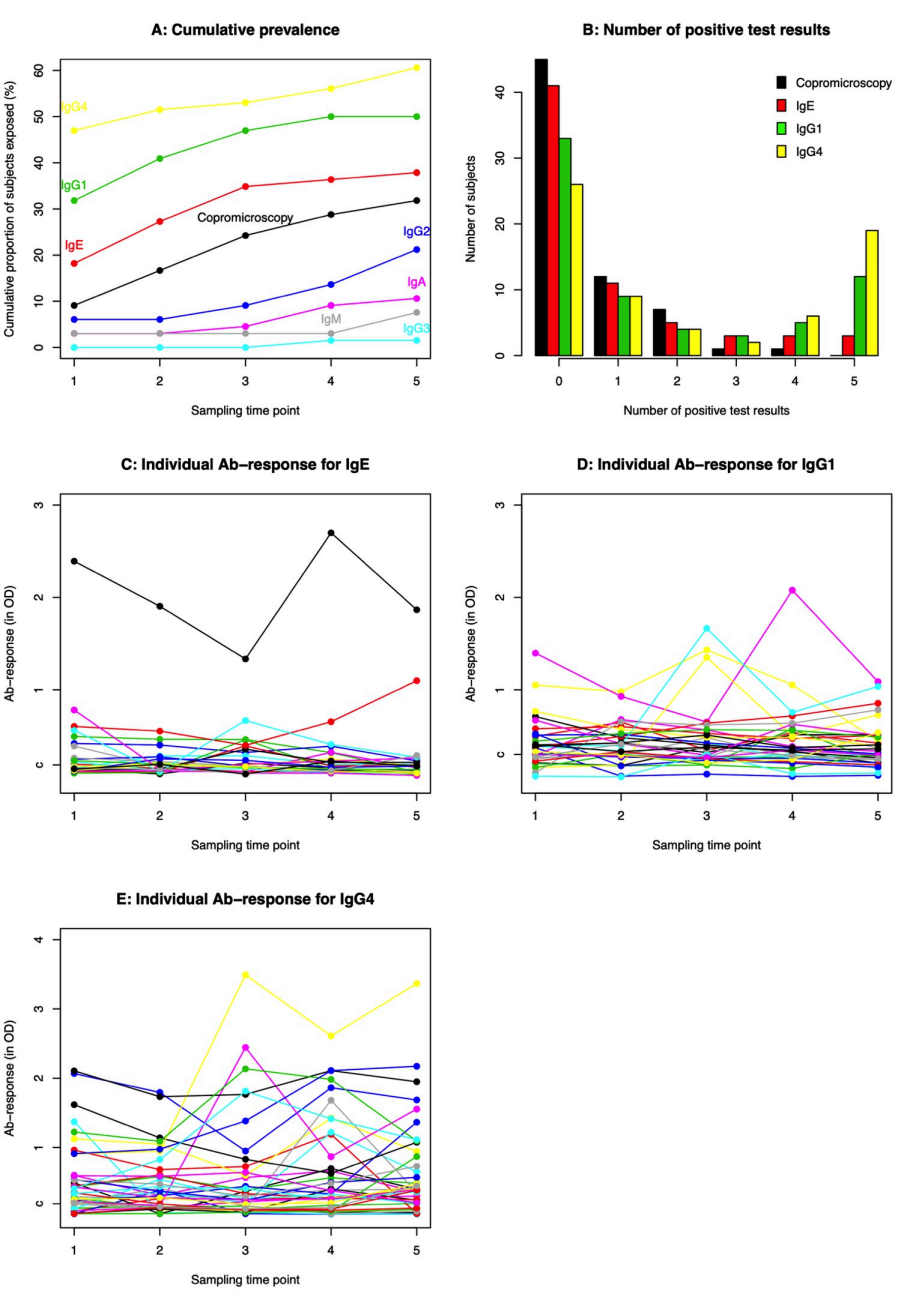
Dynamics of the anti-*Ascaris* antibody responses. The figure summarizes the dynamics of the anti-*Ascaris* antibody responses in the 66 subjects for whom complete Ab-isotype analysis was available at all five sampling points. **Panel A** illustrates the cumulative prevalence for all seven Ab-isotypes over the study period and copromicroscopy; number of positive test results for Ab-isotypes for which the cumulative prevalence exceeds that of coprology (IgE, IgG1 and IgG4) is shown in **Panel B**, the individual IgE, IgG1 and IgG4 response are illustrated in **Panels C**, **D** and **E**, respectively. The ‘c’ in **Panels C** to **E** represents the cut-off value for the corresponding Ab-ELISA. For ethical reasons, children were treated when they were positive on copromicroscopy. Large scale deworming was implemented between each sampling time points, except between sampling time point 3 and 4 (**see Fig 1**).

**Fig 5:**
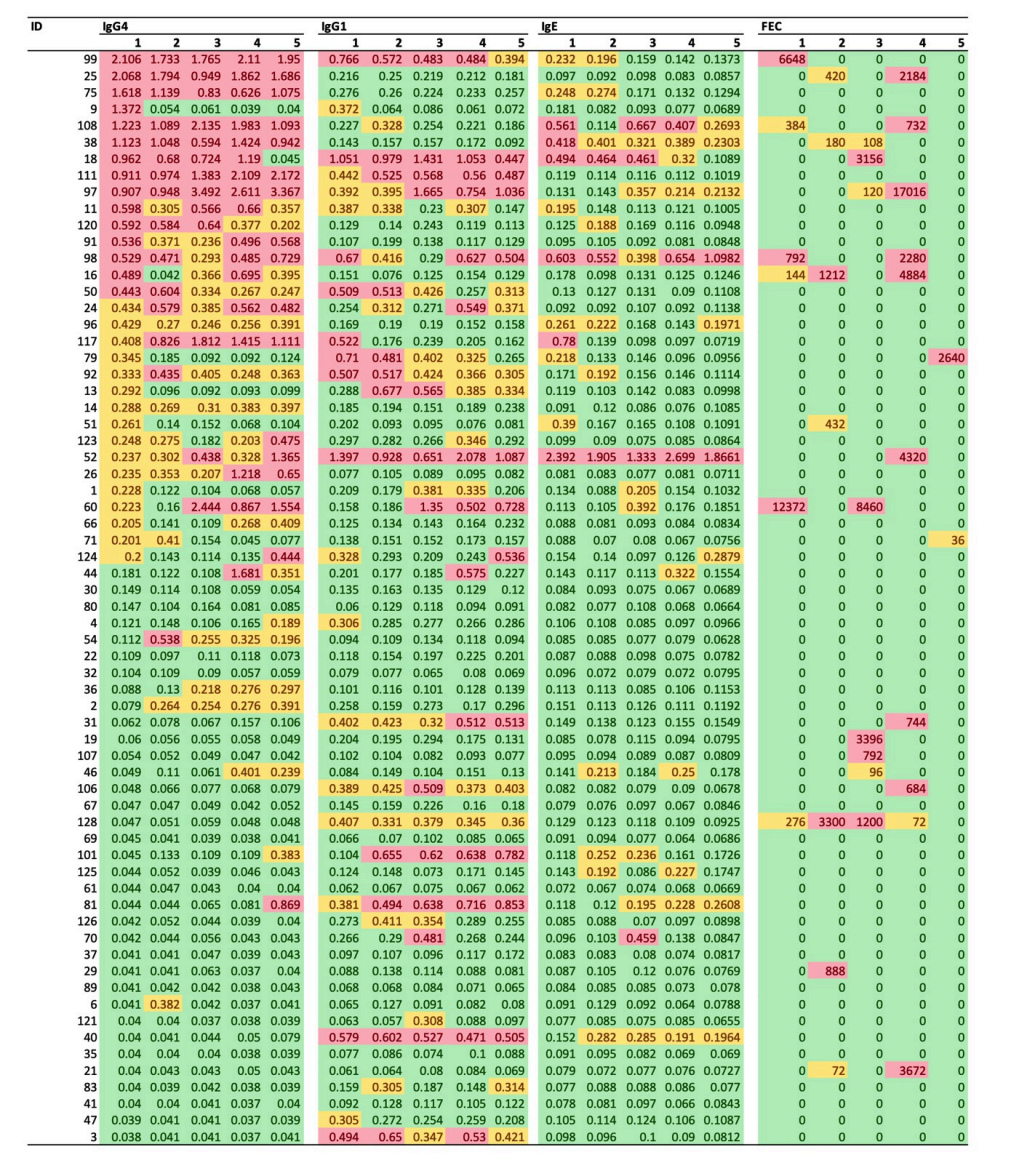
Individual *Ascaris* exposure dynamics based on three Ab-isotypes and copromicroscopy. These heat maps illustrate the dynamics of the anti-*Ascaris* IgG4, IgG1 and IgE level as well as the results of the copromicroscopy (by means of fecal egg counts (FECs, expressed as eggs per gram of stool)) over the five sampling time points. Green indicates a negative test result (Ab-ELISA: optic density (OD) less than diagnostic cut-off; copromicroscopy: FEC = 0 EPG); yellow represents a low Ab-response (decision cut-off ≤ OD < median OD across all children that tested positive at baseline) or FEC (O < FEC ≤ 588 EPG); red indicates a high Ab-response (OD ≥ median OD across all children that tested positive at baseline) or FEC > 588 EPG). The study participants were ranked based on the OD for IgG4 measured at baseline. For ethical reasons, children were treated when they were positive on copromicroscopy. Large scale deworming was implemented between each sampling time points, except between sampling time point 3 and 4 (**see Fig 1**).

The plasma samples collected at the later sampling time points were subsequently analysed with isotype specific AsLungL3 Ab-ELISAs and compared to the results obtained at baseline. As shown in **Figs 4** and **5,** the results indicate that the proportion of subjects seropositive for *Ascaris* when assessed for IgG4, IgG1 and IgE was generally higher than when measured by copromicroscopy (**Fig 4**) and that majority of the subjects already tested positive at the start of the study on at least one of the isotype specific AsLungL3 Ab-ELISAs (**Fig 5**). On the other hand, the cumulative prevalence of IgA, IgM, IgG2 and IgG3 did not exceed that of copromicroscopy. The incidence based on isotype specific AsLungL3 Ab-ELISAs was ~25.0% for IgG4 (25.7%; 9 out of 35), IgG1 (26.7%; 12 out of 25) and IgE (24.1%; 13 out of 54). For the other isotypes the incidence ranged from 1.5% (1 out of 66) for IgG3 to 16.1% for IgG2 (12 out of 45).

Interestingly, as shown in **Fig 4B**, most children who were seropositive for IgG4, IgG1 and IgE tested positive on at least two time points (14/22 for IgE, 24/33 for IgG1 and 31/40 for IgG4). These observations are also apparent in the individual test results with anti-*Ascaris* IgE, IgG1 and IgG4 levels remaining high throughout the study in several individuals (**Figs 4C, 4D**, **4E** and **5)**. A significant positive correlation in Ab-response IgG1, IgG4 and IgE was observed for each of three Ab-isotypes pairs (see **S1 Fig**, Spearman correlation coefficient between OD for IgG1 and IgG (R_IgG1-IgG4_) = 0.40; R_IgE-IgG1_: 0.60; R_IgE-IgG4_: 0.52; *p* <0.001).

### Baseline IgG4 Ab-response as a predictor for future *Ascaris* egg excretion

**Fig 6:**
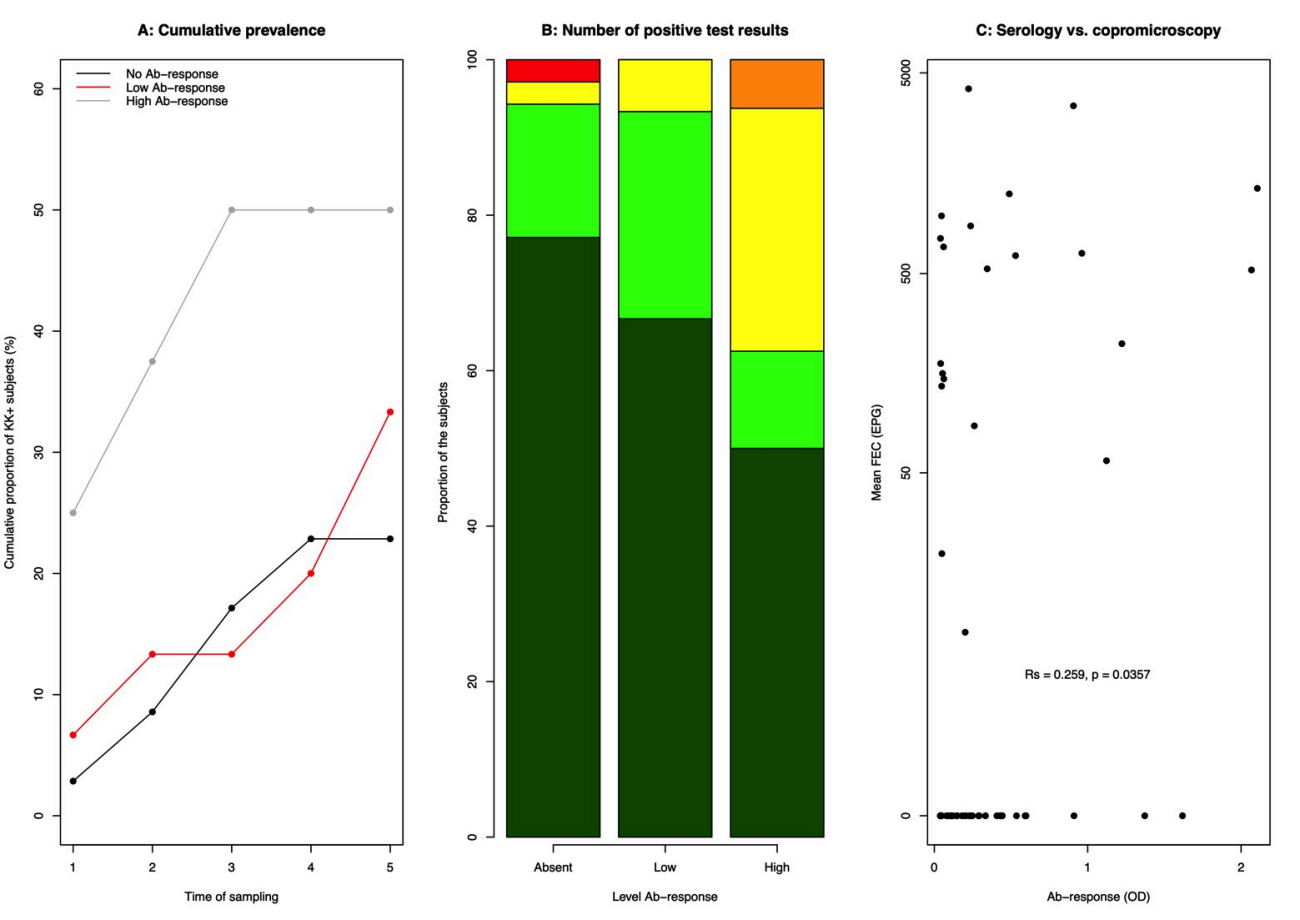
Baseline anti-*Ascaris* IgG4 levels as a predictor for future *Ascaris* egg excretion. Association between the anti-*Ascaris* IgG4 levels at the start of the study (no IgG4 response: OD < diagnostic cut-off; low IgG4 response: diagnostic cut-off ≤ OD < median OD across all children who tested positive for this Ab-isotype; high IgG4 response: OD ≥ median OD across all children who tested positive for this Ab-isotype) and the *Ascaris* cumulative prevalence based on copromicroscopy (grey line: high IgG4 response, red: low IgG4 response, black: no IgG4 response) (**Panel A**), the number of positive test results using IgG4 (0: dark green; 1: light green; 2: yellow; 3: orange; 4: red) (**Panel B**) and the mean FECs (expressed as egg counts per gram of stool (EPG)) (**Panel C**). For ethical reasons, children were treated when they were positive on copromicroscopy. Large scale deworming was implemented between each sampling time points, except between sampling time point 3 and 4 (**see Fig 1**).

Given the high number of IgG4 seropositive children at baseline, we subsequently investigated whether this parameter could be used as a predictor for *Ascaris* egg excretion later on. For this, children were categorized in three categories based on their anti-*Ascaris* IgG4 levels at baseline, i.e. no IgG4 response: OD < diagnostic cut-off; low IgG4 response: diagnostic cut-off ≤ OD < median OD across all children who tested positive for this Ab-isotype; high IgG4 response: OD ≥ median OD across all children who tested positive for this Ab-isotype. The cumulative prevalence based on copromicroscopy, the number of stool samples positive for *Ascaris* eggs and the mean *Ascaris* FECs for the three categories of children are shown in **Fig 6**. When summarizing the cumulative prevalence based on copromicroscopy for each of the three levels of IgG4 response (**Fig 6A**), it became clear that, compared to the other two IgG4 response levels, the high IgG4 response level was associated with not only a higher proportion of subjects excreting *Ascaris* eggs at the start of the study (25.0% *vs*. 6.7% *vs*. 2.9%) but also with a higher egg-appearance rate. Compared to the children with a high IgG4 response, the odds for a positive test at baseline were 0.21 (95%CI: 0.02–2.19, *p* = 0.19) and 0.09 (95%CI: 0.01–0.87, *p* = 0.03) times lower for children with a low to no IgG4 response, respectively. In the group of children with high IgG4 levels, egg-appearance rate was also higher, resulting in a steep increase in cumulative prevalence. For the children with a high IgG4 response, the cumulative prevalence reached its maximum (50.0%) at the third sampling point, where for the other two Ab-response categories the highest value (no IgG4 response: 22.9% and low IgG4 response: 30.0%) was only observed towards the end of the study. In addition to this, the children with a high IgG4 response were also more likely to have more positive test results during the course of the study (**Fig 6B**). Six out of the 16 (37.5%) subjects with high IgG4 levels tested positive at least two times, whereas for the subjects with a low or no IgG4 response, these numbers were 1 out of 15 (6.7%) and 2 out 35 (5.7%), respectively. Compared to the children with a high IgG4 response, the odds for minimum two positive test results were 0.12 (95%CI: 0.01–1.15, *p* = 0.07) and 0.10 (95%CI: 0.02–0.58, *p* = 0.01) times lower for children with a low or no IgG4 response, respectively. Finally, a significant positive correlation between the individual anti-*Ascaris* IgG4 levels at baseline and the mean FEC across the five sampling time points was observed (Rs = 0.259, *p* = 0.036) (**Fig 6C**).

### Assessment of the cross reactivity of *Trichuris* on the *Ascaris* IgG4 ELISA

In order to explore potential cross reactivity between *Trichuris* and the anti-*Ascaris* IgG4 Ab-ELISA, we assessed the associations between the anti-*Ascaris* IgG4 levels at the start of the study and copromicroscopy for *Trichuris*, excluding those children that tested positive at copromicroscopy at any of the sample time points. Due to the low number of hookworm infections that did not test positive for *Ascaris* by copromicroscopy (n = 5), cross-reactivity with this STH was not explored.

**Fig 7:**
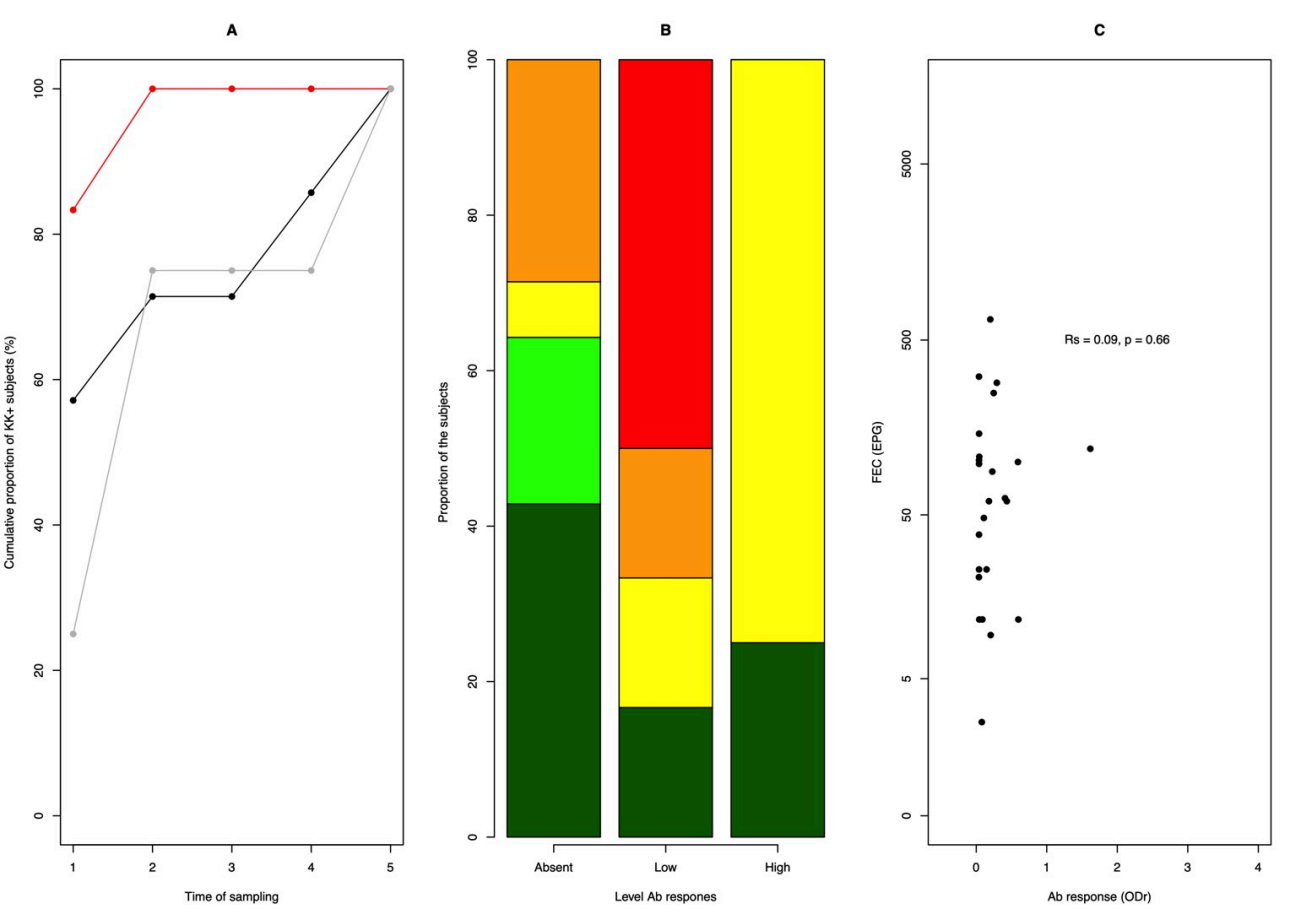
Analysis of the potential cross-reactivity due to *Trichuris* on anti-*Ascaris* IgG4 levels. The figure illustrates the association between the anti-*Ascaris* IgG4 levels (no IgG4 response: OD < diagnostic cut-off; low IgG4 response: diagnostic cut-off ≤ OD < median OD across all children who tested positive for this Ab-isotype; high IgG4 response: OD ≥ median OD across all children who tested positive for this Ab-isotype) and the *Trichuris* cumulative prevalence based on copromicroscopy (grey line: high IgG4 response, red: low IgG4 response, black: no IgG4 response) (**Panel A**), the number of IgG4 positive test results (0: dark green; 1: light green; 2: yellow; 3: orange; 4: red; 5: dark red) (**Panel B**) and the mean FECs (expressed as egg counts per gram of stool (EPG) (**Panel C**). In this graph we only included subjects that did not test positive at copromicroscopy at any time point, but that tested at least once on *Trichuris* (n = 26). For ethical reasons, children were treated when they were positive on copromicroscopy. Large scale deworming was implemented between each sampling time points, except between sampling time point 3 and 4 (**see Fig 1**).

The results are summarized in **Fig 7**, and did not indicate any apparent cross-reactivity of *Trichuris* with anti-*Ascaris* IgG4. In contrast to the observations for *Ascaris*, the highest proportion of children excreting eggs of *Trichuris* was observed in those with a moderate IgG4 response (5 out of 6, 83.3%), the lowest proportion was observed with the children with the highest IgG4 response (1 out of 4, 25.0%). The cumulative prevalence was 100% for each of the three levels of Ab response (**Fig 7A**). Based on the number of positive samples, 3 out of the 4 (75.0%) study subjects with high IgG4 response were excreting the eggs of *Trichuris* more than two times, whereas for the subjects with a low or no IgG4 response these numbers were 5 out of 14 (35.7%) and 5 out 6 (83.3%), respectively (**Fig 7B**). Finally, there was no significant positive correlation between the individual anti-*Ascaris* IgG4 response at baseline and the mean FEC of *Trichuris* across the five sampling time points (Rs = 0.09, *p* = 0.66) (**Fig 7C**).

## Discussion

### Children continue to be exposed to STH from an early age onwards

At the start of this study, more than 40% of the children (age 4–6 years of age) were excreting the eggs of any STH, highlighting that children are already exposed to STH infections at an early age. These findings are in line with other studies reporting high STH prevalence in these age groups [18,23–25] and underscore the need to continue supporting efforts to include pre-school aged children in national deworming programs. According to the PC coverage report of WHO, Ethiopia reached 66.9% of the pre-school aged children at risk of the STH-attributable morbidity [26]. The follow-up of the children also indicated that children are continuously exposed to STH, despite the periodical administration of anthelmintic drugs as part of either this study (21 out of the 66 children received 400 mg albendazole) or the national school-based deworming program (four rounds, though we could not always confirm the compliance of our cohort). At the end of the study, 72.7% of the children tested positive at least once for any STH, though important differences can be noted across STHs. Whereas, the cumulative prevalence tripled and doubled for Ascaris (9.1% to 31.8%) and hookworms (4.5% to 9.1%) respectively, it increased from 37.9% to 56.1% for *Trichuris*. In addition to this, re-infections were frequently observed throughout the study period underscoring the need for additional control measures (improved water, sanitation and hygiene and health education) to supplement the deworming programs. This is particularly important as recent research indicates the presence of the different life stages (egg and larvae) of a variety of helminth species (e.g., STHs, *Taenia* spp, and *Hymenolepis* spp) in the environment of the schools (playground, class rooms and latrines), the households and the open markets in Jimma Town. Furthermore, for *Trichuris*, there is also a need for more efficacious treatments, either through more potent drugs (oxantel) or drug combinations (albendazole and oxantel) [27], as 9% of the subjects were excreting eggs at each of the five sampling time points of the study.

### Anti-*Ascaris* antibody levels indicate that more children are exposed to *Ascaris* than detected by copromicroscopy

The results of the anti-*Ascaris* IgG4 Ab-ELISA confirmed that children are already exposed to *Ascaris* at an early age, but also indicated that proportionally more children are exposed to *Ascaris* when measured by serology (IgG4: 47%; IgG1: 31.8% and IgE: 18.2%) than through copromicroscopy (9.1%). This discrepancy in prevalence between both diagnostic methods is in line with previous studies in both animals [9,28] and humans [13,14], and can be explained by the natural expulsion of immature larval stages by infected individuals. In other words, not all immature worms will eventually grow into adult worms, and hence the number of adult worms in the intestine will not be representative for the level of exposure to the infectious eggs [29,30]. As a consequence of this, the disease burden of ascariasis in humans is likely to be underestimated. Indeed, as shown in animal models, the impact of migrating immature larvae on the liver and lung is substantial [26,31]. Where serology was associated with production parameters in animals, it remains unclear whether serology would provide a more reliable tool for assessing morbidity attributable to *Ascaris* infections in humans.

### Highest seroconversion against *Ascaris* measurable for IgG4

Analysis of the antibody responses indicated that mainly the anti-*Ascaris* IgG and IgE levels were elevated in the study population and in particular IgG4. This finding is in line with previous studies both for *Ascaris* and other helminth infections, such as strongyloidiasis [32], filariasis [33], onchocerciasis [34], toxocariasis [35] and schistosomiasis [36]. IgG4 antibodies have shown to be typically formed during repeated or long-term infections and exposure to antigen [37], which fits with the typical chronic and repeated character of worm infections. Importantly, IgG4 subclass switching has shown to be modulated by IL-10 which is also important for the control and downregulation of immune responses. Hence, it has been suggested that high IgG4 levels in worm-infected individuals are indicative of a regulatory immune environment thereby protecting the individual for potentially damaging inflammation, this in contrast with IgE [38]. Interestingly, although anti-AsLungL3 IgG4 levels were in general positively correlated with IgE levels, a limited number of individuals predominantly showed an IgE response. If and how the ratio IgG4/IgE changes over time in these individuals is still unclear and requires further research.

### IgG4 response as a predictor for future *Ascaris* egg excretion

Our results highlighted an association between *anti-Ascaris* IgG4 levels at baseline and the *Ascaris* egg excretion measured by copromicroscopy. The individual IgG4 levels at baseline were positively associated with the FECs averaged over the study period, the rate of egg-appearance and the number of positive test results. These associations once more highlight the potency of Ab-ELISA as an additional tool to improve STH control programs. This is because it allows for more targeted distribution of drugs to those individuals that are at higher risk for re-infection. The assay can also be deployed to gain insights into why these children continuously re-infect themselves. To be more concrete, deployment of this Ab-ELISA would allow assessing differences in behavioural, socio-economic and environmental factors between children with a high Ab-response and those with a low Ab-response.

The associations between the anti-*Ascaris* IgG4 responses and *Trichuris* infections based on copromicroscopy were also analysed and suggested that there is no or a limited level of cross-reactivity. This finding is in line with other studies reporting lack of cross-reactivity of anti-*Ascaris* IgG4 with other nematodes [39,40]. Although, this finding is in line with our previous work (including *Toxoxara*; [14]), these conclusions might be flawed by the small number of cases that were *Ascaris* negative based on copromicroscopy any sampling time point but that tested positive at least once for *Trichuris* (n = 26) or hookworm (n = 5).

### Road towards the implementation of serology in STH control programs

Although our findings further highlighted that our Ab-ELISA holds promise as a tool to inform STH control programs, much more information still needs to be produced before it can be implemented. One of the most important outstanding issues is the assessment of the diagnostic performance (clinical sensitivity and specificity) of our Ab-ELISA to detect *Ascaris* infections in absence of a gold standard. Although WHO recently published the minimal and ideal requirements–the so-called target product profiles (TPPs)–for diagnostics to monitor and evaluate STH control programs [41], the working group that developed these TPPs did not feel comfortable with putting forward a comparator. For completeness, we summarized the agreement in test results between copromicroscopy and the Ab-ELISA in **S1 Table,** highlighting that at baseline both tests agreed in 39 (5 positive test results and 34 negative test results) out of the 66 subjects. In the 27 remaining cases, 26 tested positive on Ab-ELISA but not on copromicroscopy. A single case tested positive on copromicroscopy but not on Ab-ELISA. These discrepancies in qualitative test results between diagnostic methods are not new and were even more extreme in our previous cross-sectional study in the same study area [14], where ~44% of the subjects that tested positive on copromicroscopy were seronegative, and ~80% of the seropositive subjects did not excrete eggs. These more extreme discrepancies can be explained by the different age groups that were examined in both studies. While the present study focussed on school children of (4–6 years of age), our previous study involved both children (5–10 years: 300; 14–17 years: 263) and adults (18–85: 637). In contrast to the younger age groups where agreement in qualitative test results was relatively high (5–10 years: 48.3%; 14–17 years: 43.7%), this was poor in adults (22.4%). This poor agreement in both tests in adults may be the result of an elevated protective immune response due to continued *Ascaris* exposure that prevents the development of new patent infections [42,43].

Similarly, it is apparent that the associations between quantitative test results is probably too weak to establish Ab-ELISA-specific thresholds for moderate-to-heavy intensity infections, as currently defined by copromicroscopy (FEC ≥5,000 EPG [20]), in children (**Fig 6**). However, here too one could question whether an egg-based measure is the most appropriate measure for the true underlying burden caused by *Ascaris* (e.g., infections with adult males only and tissue damage caused by migrating larvae). For example, experiments in animal models highlighted an association between the quantitative test results of our Ab-ELISA and the number of white spots on the liver (damage caused by migrating larvae) [10,28] in absence of any adult worm, once more underscoring the pivotal role of animal models to gain insights into the diagnostic performance of new diagnostic tests. Particularly pigs offer a great infection model as they can be artificially infected with a single STH species (*Ascaris suum*) and can be followed throughout the life cycle, resembling the ultimate gold standard.

## Conclusion

Our results underscore that children are exposed to STHs from an early age onwards and that significantly more children seem to be exposed to *Ascaris* based on serology than initially measured by copromicroscopy. Moreover, IgG4 is the Ab-isotype of choice to measure exposure to *Ascaris* using the AsLungL3 Ab-ELISA. Furthermore, this study highlights that Ab-ELISAs based on IgG4 hold promise as a tool to identify individuals at higher high risk for continued exposure to worm infections, which is important information to further improve control programs.

## Supporting information

S1 Fig The correlation in Ab-response between IgG4, IgG1 and IgE. These scatter plots represent the correlation in Ab-response (measured as optical density (OD)) between IgG4, IgG1 and IgE. The red lines represent the diagnostic cut-off of each Ab-ELISA, the red numbers in the quadrant represent the agreement in test results (**bottom left quadrant**: both Ab-ELISAs tested negative; **top right quadrant**: both Ab-ELISAs tested positive). The Spearman correlation coefficient (Rs) and the corresponding level of significance is provided in the top right corner.
(TIF)

S1 Info The details on the secondary antibodies used in the iso-type specific Ab-ELISA. (DOCX)

S1 Table The agreement in test results between copromicroscopy and anti-AsLungL3 Ab-ELISA. (DOCX)

S1 Dataset The data set includes the plasma antibody (Ab-)responses against the total extract of *Ascaris suum* lung third stage larvae (AsLungL3) as measured through in-house Ab-ELISA’s for seven different Ab-isotypes (IgA, IgE, IgM and IgG sub-classes (IgG1, IgG2, IgG3 and IgG4) (‘Ab-ELISA’-tab), the fecal egg counts (in eggs per gram of stool) based on duplicate Kato-Katz thick smear (‘Duplicate Kato-Katz’-tab) and the analysis to determine the diagnostic cut-off for each of the Ab-ELISAs (‘Determine diagnostic cut-off’-tab).
(XLSX)
