# Supplementary figures and images for: Longitudinal assessment of the exposure to Ascaris lumbricoides through copromicroscopy and serology in school children from Jimma Town, Ethiopia
Source: PLoS Negl Trop Dis. 2022 Jan 18;16(1):e0010131. doi: 10.1371/journal.pntd.0010131 (PMC8797258; doi:10.1371/journal.pntd.0010131)

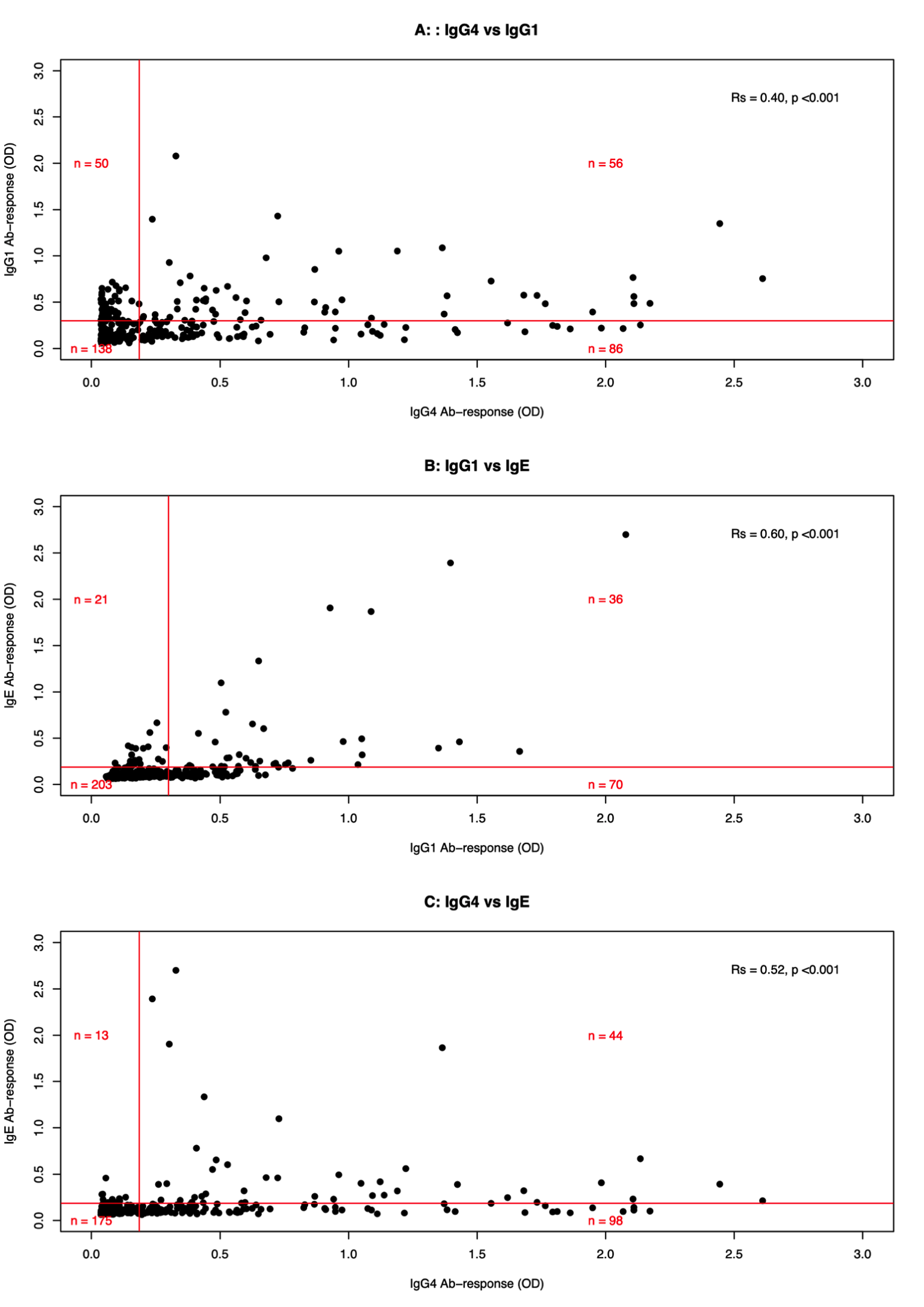

Supplement: S1 Fig — These scatter plots represent the correlation in Ab-response (measured as optical density (OD)) between IgG4, IgG1 and IgE. The red lines represent the diagnostic cut-off of each Ab-ELISA, the red numbers in the quadrant represent the agreement in test results (bottom left quadrant: both Ab-ELISAs tested negative; top right quadrant: both Ab-ELISAs tested positive). The Spearman correlation coefficient (Rs) and the corresponding level of significance is provided in the top right corner. (TIF) [file pntd.0010131.s001.tif]
