# Supplementary material for: Longitudinal assessment of the exposure to Ascaris lumbricoides through copromicroscopy and serology in school children from Jimma Town, Ethiopia
Source: PLoS Negl Trop Dis. 2022 Jan 18;16(1):e0010131. doi: 10.1371/journal.pntd.0010131 (PMC8797258; doi:10.1371/journal.pntd.0010131)
Supplement: S1 Info — (DOCX) [file pntd.0010131.s002.docx]

**Longitudinal assessment of the exposure to *Ascaris lumbricoides* through copromicroscopy and serology in school children from Jimma Town, Ethiopia**

**Running title:** Longitudinal assessment of *Ascaris* infections in school children

Daniel Dana^1,2*^, Sara Roose^2^, Johnny Vlaminck^2^, Mio Ayana^1,2^, Zeleke Mekonnen^1^, Peter Geldhof^2^, Bruno Levecke^2*^

^1^School of Laboratory Science, Faculty of Health Science, Institute of Health Jimma University, Jimma, Ethiopia

^2^Department of Translational Physiology, Infectiology and Public Health, Ghent University, Merelbeke, Belgium

*****danidana2000@gmail.com (DD); *bruno.levecke@ugent.be (BL)

**S1 Info. The details on the secondary antibodies used in the iso-type specific Ab-ELISA.**

| **Product** | **Cat. No.** | **Link** |
| --- | --- | --- |
| Mouse anti-human IgG1 Fc Secondary Antibody, HRP | A-10648 | <https://www.thermofisher.com/antibody/product/Mouse-anti-Human-IgG1-Fc-Secondary-Antibody-Monoclonal/A-10648> |
| Mouse anti-Human IgG2 Secondary Antibody, HRP | MH1722 | <https://www.thermofisher.com/antibody/product/Mouse-anti-Human-IgG2-Secondary-Antibody-Monoclonal/MH1722> |
| Mouse anti-Human IgG3 (Hinge) Secondary Antibody, HRP | 05-3620 | <https://www.thermofisher.com/antibody/product/Mouse-anti-Human-IgG3-Hinge-Secondary-Antibody-Monoclonal/05-3620> |
| Mouse Anti-Human IgG4 pFc’-HRP | 9190-05 | https://www.southernbiotech.com/?catno=9190-05&type=Monoclonal#&panel2-1 |
| Mouse anti-Human IgM (Heavy chain) Secondary Antibody, HRP | 05-4920 | <https://www.thermofisher.com/antibody/product/Mouse-anti-Human-IgM-Heavy-chain-Secondary-Antibody-Monoclonal/05-4920> |
| Goat anti-Human IgA Secondary Antibody, HRP | A18781 | <https://www.thermofisher.com/antibody/product/Goat-anti-Human-IgA-Secondary-Antibody-Polyclonal/A18781> |
| Mouse anti-Human IgE Secondary Antibody, HRP | SA5-10306 | <https://www.thermofisher.com/antibody/product/Mouse-anti-Human-IgE-Secondary-Antibody-Monoclonal/SA5-10306> |
