# Supplementary material for: Longitudinal assessment of the exposure to Ascaris lumbricoides through copromicroscopy and serology in school children from Jimma Town, Ethiopia
Source: PLoS Negl Trop Dis. 2022 Jan 18;16(1):e0010131. doi: 10.1371/journal.pntd.0010131 (PMC8797258; doi:10.1371/journal.pntd.0010131)
Supplement: S1 Table — (DOCX) [file pntd.0010131.s003.docx]

**Longitudinal assessment of the exposure to *Ascaris lumbricoides* through copromicroscopy and serology in school children from Jimma Town, Ethiopia**

**Running title:** Longitudinal assessment of *Ascaris* infections in school children

Daniel Dana^1,2*^, Sara Roose^2^, Johnny Vlaminck^2^, Mio Ayana^1,2^, Zeleke Mekonnen^1^, Peter Geldhof^2^, Bruno Levecke^2*^

^1^School of Laboratory Science, Faculty of Health Science, Institute of Health Jimma University, Jimma, Ethiopia

^2^Department of Translational Physiology, Infectiology and Public Health, Ghent University, Merelbeke, Belgium

*danidana2000@gmail.com (DD); *bruno.levecke@ugent.be (BL)

**S1. Table. The agreement in test results between copromicroscopy and anti-AsLungL3 Ab-ELISA.**

|  | **Sample time point 1** | | |  |  | **Sample time point 2** | | |  |
| --- | --- | --- | --- | --- | --- | --- | --- | --- | --- |
|  |  |  | Copromicroscopy |  |  |  |  | Copromicroscopy |  |
|  |  |  | - | + |  |  |  | - | + |
|  | Ab-ELISA | - | 34 | 1 |  | Ab-ELISA | - | 36 | 5 |
|  |  | + | 26 | 5 |  |  | + | 23 | 2 |
|  |  |  |  |  |  |  |  |  |  |
|  | **Sample time point 3** | | |  |  | **Sample time point 4** | | |  |
|  |  |  | Copromicroscopy |  |  |  |  | Copromicroscopy |  |
|  |  |  | - | + |  |  |  | - | + |
|  | Ab-ELISA | - | 37 | 4 |  | Ab-ELISA | - | 33 | 4 |
|  |  | + | 21 | 4 |  |  | + | 23 | 6 |
|  |  |  |  |  |  |  |  |  |  |
|  | **Sample time point 5** | | |  |  | **All 5 sample time points** | | |  |
|  |  |  | Copromicroscopy |  |  |  |  | Copromicroscopy |  |
|  |  |  | - | + |  |  |  | - | + |
|  | Ab-ELISA | - | 32 | 2 |  | Ab-ELISA | - | 172 | 16 |
|  |  | + | 32 | 0 |  |  | + | 125 | 17 |
|  |  |  |  |  |  |  |  |  |  |
